# Measles seroprevalence among health care professionals during a period of increased transmission: A pilot study in Southern Italy

**DOI:** 10.1016/j.ijregi.2025.100833

**Published:** 2025-12-27

**Authors:** Davide Anzà, Vincenzo Restivo, Francesca Cirillo, Bruna Lo Sasso, Begoña Martínez-Jarreta, Paola Senia, Ermanno Vitale

**Affiliations:** 1 Department of Medicine and Surgery, University of Enna “Kore,” Enna, Italy; 2 Local Health Unit of Enna, Enna, Italy; 3 Forensic and Occupational Medicine, Department of Pathological Anatomy, Forensic and Legal Medicine and Toxicology, University of Zaragoza, Zaragoza, Spain; 4 Local Health Unit of Catania, Catania, Italy

**Keywords:** Health care professionals, Immunity, Measles, Occupational epidemiology, Vaccine

## Abstract

**Objectives:**

Measles continues to be a significant public health concern worldwide, with suboptimal vaccination coverage contributing to periodic outbreaks, even in Europe. Despite the availability of an effective vaccine, regional disparities and declining vaccination coverage, exacerbated by the COVID-19 pandemic, have increased the risk of measles transmission in Italy.

**Methods:**

This study aimed to assess measles seroprevalence among health care professionals (HCPs) and explore associated socio-demographic factors in a country with a high level of measles circulation.

**Results:**

Data were collected from 148 HCPs across two hospitals in Southern Italy. Serologic analyses were performed on 121 of the 148 enrolled HCPs, revealing an overall seroprevalence of 96%; however, there was a substantial proportion of non-immune young female HCPs. Furthermore, smoking was significantly associated with reduced immunoglobulin levels (adjusted odds ratio = 13.3; 95% confidence interval = 1.07-166.2; *P* <0.04).

**Conclusions:**

These findings underscore the importance of routine serological monitoring among HCPs to inform booster vaccination strategies to fill immunity gaps in high-risk groups and settings.

## Highlights

• High measles seroprevalence (96%) observed among health care professionals.
• Younger female health care professionals showed the greatest immunity gaps.
• Smoking status was independently associated with reduced immunity.
• No significant differences across gender, body mass index, or occupation.
• Findings highlight the need for targeted vaccination strategies.

## Introduction

Despite being vaccine-preventable, measles remains a leading global cause of morbidity and mortality, with an estimated 10,341,000 cases and over 107,500 deaths reported worldwide in 2023 [1]. Given its high contagiousness (R₀: 12-18) and potential for severe complications, such as pneumonia, encephalitis, and death, maintaining high vaccination coverage is essential to ensure effective prevention and control [2,3]. The two-dose schedule of the measles-mumps-rubella (MMR) vaccine—started as early as 9-12 months—is highly effective and necessary to achieve herd immunity, which requires at least 95% coverage [[3], [4], [5]]. Nevertheless, measles outbreaks frequently expose inequalities in health care access and highlight gaps in immunization strategies and primary care systems [6]. In Italy, the MMR vaccine has been available since 1987 and was made mandatory for school attendance under Law No. 119 of July 31, 2017 [7]. However, global and national vaccination coverage remain suboptimal, with regional disparities in Italy, particularly, in the uptake of the second dose [8]. In 2023, MMR coverage in Italy was 94.6% for the first dose at 24 months but declined to 84.7% at 5-6 years and 89.7% at 18 years. In Sicily, the coverage was even lower: 90.8% at 24 months, 71.0% at 5-6 years, and 77.1% at 18 years [8]. Notably, natural infection provides lifelong immunity, whereas waning immunity in vaccinated individuals may contribute to sporadic cases even in highly vaccinated populations [9,10].

The COVID-19 pandemic has disrupted immunization programs, exacerbating immunity gaps and delaying routine vaccinations, which, together with the easing of containment measures, has contributed to a global resurgence of measles [6,11,12]. In the United States, the Centers for Disease Control and Prevention reported an increase from 285 in 2024 to 1,046 in early 2025 [13].

In the European Union and European Economic Area measles incidence rose sharply after the COVID-19 pandemic, reaching 5.2 cases per million in 2023 vs 0.3 cases per million in 2022 [14]. In Italy, the Italian National Institute of Health reported 18.3 cases per million in 2024, with Sicily showing one of the highest burdens (35.5 cases per million) [3]. In January 2025 alone, national cases already exceeded those of the same month in 2024, underscoring the need to strengthen vaccination and surveillance systems [3].

Health care professionals (HCPs) play a pivotal role in preventing measles transmission. They are at an elevated risk of infection and can transmit the virus to vulnerable patients [15]. Despite high immunization rates reported among HCPs in Italy, the coverage levels in this group often fall short of the recommended threshold of >95%. Indeed, they represented a significant proportion of measles cases in Italy in 2022, accounting for 33.3% of the total reported cases [3,16]. Past hospital outbreaks involving HCPs show that hygiene and isolation alone are insufficient without vaccination [17]. Therefore, the Italian Society of Occupational Medicine recommends administering two doses of the MMR vaccine [18].

The Italian National Vaccination Prevention Plan (PNPV) 2023-2025 identifies strengthening vaccination coverage as a strategic priority [5]. Among its key initiatives, the plan emphasizes improving public communication on vaccination benefits, increasing awareness among HCPs, and providing targeted training in vaccinology [5]. However, the effectiveness of these strategies hinges on the capacity to identify and address immunity gaps across population subgroups.

This study aimed to conduct a seroprevalence analysis to evaluate the immunization status against measles of HCPs of two hospitals in southern Italy, during a period of high viral circulation. Furthermore, any possible association between risk factors and habits associated with the immunization status of HCPs was evaluated.

## Materials and methods

### Study design

This study involved a cohort of HCPs—including physicians, nurses, laboratory technicians, healthcare assistants, and social health workers—from Umberto I Hospital (Enna) and M. Chiello Hospital (Piazza Armerina) between March and September 2024, as part of mandatory occupational surveillance. Recruitment was facilitated by the Occupational Medicine Service of the Enna Local Health Unit. All participants undergoing routine medical checkups, such as hematologic assessments, were invited to participate in anti-measles immunoglobulin (Ig) G testing, providing a representative evaluation of measles immunity within the hospital workforce.

### Sample size

The sample size was determined based on an expected 86% positive attitude among HCPs toward vaccination with the measles vaccine, with a 95% confidence interval (CI) and a 5% margin of error. Assuming a response rate of 80%, the final required sample size was calculated to be 141.

### Serologic analysis

Blood samples were analyzed at Umberto I Hospital’s Clinical Pathology Laboratory. After centrifugation at 4000 rpm for 10 minutes, serum IgG titers were measured using an enzyme-linked immunosorbent assay kit (Eurospital Diagnostics). Titers >11 IU/ml indicated immunity, with the assay showing 99.2% sensitivity and 98.6% specificity.

### Questionnaire

A questionnaire was administered to recruited HCPs, who received detailed information on the study objectives and data confidentiality. It included three sections: the first collected demographic data (age, sex, weight, height, job role, department, smoking, and alcohol habits); the second gathered clinical information (comorbidities, drug use, measles antibody levels, and vaccination history); the third assessed measles vaccination acceptance using the health belief model (HBM). The HBM evaluated four domains: perceived susceptibility, perceived severity, perceived barriers, and perceived benefits of vaccination. Responses were recorded on a five-point Likert scale, from “strongly agree” to “strongly disagree,” and then converted to an ordinal scale, with 1 representing “strongly disagree” and 5 representing “strongly agree” (Table 1).

**Table 1:** Health belief model items on measles vaccination acceptance among HCPs.

Perceived susceptibility	a) HCPs are at increased risk of contracting measles due to occupational exposure. b) Measles can be easily transmitted in healthcare settings through contact with infected patients or colleagues.
Perceived severity	c) Measles can lead to severe complications (e.g. pneumonia, encephalitis) and may require hospitalization. d) Measles infection in HCPs can result in work absenteeism and transmission to vulnerable patients.
Perceived barriers	e) I believe that I can easily access vaccination services to receive the MMR vaccine. f) Concerns about vaccine safety or side effects may discourage HCPs from receiving the MMR vaccine.
Perceived benefits	g) The MMR vaccine is effective in preventing measles infection. h) The MMR vaccine has a favorable safety profile and does not cause severe adverse reactions.

HCP, health care professional; MMR, measles-mumps-rubella.

### Statistical analysis

The normality of quantitative variables was assessed using the skewness and kurtosis test. Normally distributed variables were expressed as mean (SD), whereas non-normal variables as median (interquartile range [IQR]). Qualitative variables were summarized as absolute and relative frequencies. Associations between quantitative variables and measles immunization were analyzed using Student’s *t*-test or Wilcoxon and Mann–Whitney tests, and chi-square tests were applied for qualitative variables. Variables significantly associated with lack of immunization were included in a forward stepwise multivariate logistic regression model using the likelihood ratio test. All statistical analyses were performed using STATA MP v14.2, ensuring the reliability and robustness of the results.

## Results

### Descriptive analysis

A total of 148 HCPs participated in the study. Most were female (72%, n = 107), with a mean age of 49.1 years (SD = 11). Nurses represented 51% of participants, followed by physicians (21%). Regarding health characteristics and habits, 30.4% were overweight and 16.9% were obese; 32.4% reported comorbidities, 39.9% regularly used medications, 25.7% were smokers, and 50.7% consumed alcohol at least once per month.

Regarding immunity against measles, 75.6% of participants reported past infection and 13.5% had been vaccinated. Among the 121 HCPs with known immunization status, 95.3% of females and 97.5% of males were immune. Overall, 4% (n = 5) were non-immune, mainly, young women aged 30-33 years, and 60% of unvaccinated HCPs were aged under 35 years. No significant differences in immunity were observed by gender (P = 0.35) or professional category (P = 0.16) (Table 2, Table 3).

**Table 2:** Socio-demographic information of the HCPs.

	Total (n = 148)^a^	Health care workers immunized against measles (n = 116, 95.9%)	Health care workers not immunized against measles (n=5, 4.1%)	*P*
Gender^b^				
Male	41 (28%)	29 (25%)	1 (20%)	0.8
Female	107 (72%)	87 (75%)	4 (80%)	
Mean age (SD)	49.1 (11.0)	49.1 (11.1)	49.7 (10.2)	0.82
Profession				
Physician	31 (21%)	23 (19.83%)	2 (40%)	0.82
Nurse	76 (51%)	59 (50.86%)	2 (40%)	
HCP	16 (11%)	14 (12.07%)	1 (20%)	
Healthcare assistant	22 (15%)	18 (15.52%)	0 (0%)	
Medical residents	1 (1%)	1 (0.86%)	0 (0%)	
Other	2 (1%)	1 (0.86%)	0 (0%)	
Years of work experience				
<5	20 (14%)	17 (14.66%)	2 (40%)	0.46
5-10	25 (17%)	22 (18.97%)	1 (20%)	
>10	29 (20%)	28 (24.14%)	1 (20%)	
>20	74 (50%)	49 (42.24%)	1 (20%)	
Recommended vaccination for others				
Yes	93 (65.5%)	76 (65.52%)	4 (80%)	0.47
No	16 (11.3%)	14 (12.07%)	1 (20%)	
No opinion	33 (23.2%)	25 (21.55%)	0 (0%)	

HCP, health care professional.

a Among the 148 HCPs recruited, 27 declined to undergo blood sampling for serological analysis.

b Information about the sex of the recruited individuals was collected through the National Health System registers and subsequently verified by the self-reported information of the participants during recruitment.

**Table 3:** Clinical information and lifestyle habits of HCPs.

	Total (n = 148)^a^	Health care workers immunized against measles (n = 116, 95.9%)	Health care workers not immunized against measles (n = 5, 4.1%)	*P*
Body mass index (kg/m^2^)				
Underweight	2 (1.4%)	2 (1.72%)	0 (0%)	
Normal weight	75 (50.7%)	59 (50.86%)	4 (80%)	
Overweight	45 (30.4%)	34 (29%)	0 (0%)	0.66
Type I obesity	25 (16.9%)	20 (17.24%)	1 (20%)	
Type II obesity	1 (0.7%)	1 (0.86%)	0 (0%)	
Comorbidity				
Yes	48 (32.4%)	36 (31.03%)	2 (40%)	0.67
No	100 (67.6%)	80 (68.97%)	3 (60%)	
Drugs consumption				
Yes	59 (39.9%)	9 (7.76%)	0 (0%)	0.52
No	89 (60.1%)	107 (92.24%)	5 (100%)	
Smoking habit				
Never smoked	91 (61.5%)	75 (64.66%)	2 (40%)	0.1
Quit smoking >10 years	15 (10.1%)	10 (8.62%)	0 (0%)	
Quit smoking <10 years	4 (2.7%)	3 (2.59%)	0 (0%)	
Active smoker	38 (25.7%)	28 (24.14%)	3 (60%)	
At least one alcohol unit per month				
Yes	75 (50.7%)	60 (51.72%)	4 (%)	0.25
No	73 (49.3%)	56 (48.28%)	1 (%)	

HCP, health care professional.

a Among the 148 HCPs recruited, 27 declined to undergo blood sampling for serological analysis.

**Figure 1:**
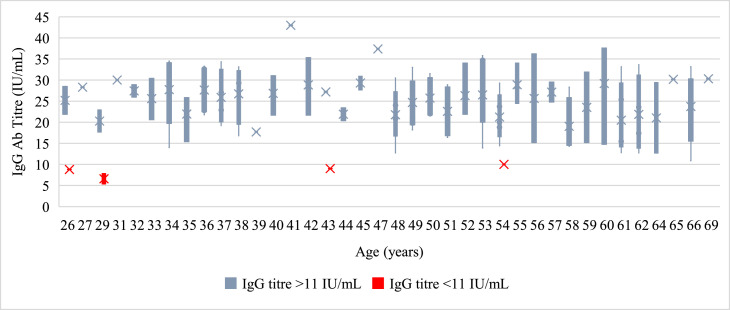
1Box plot with measles IgG antibody index by age for health care workers. Ab, antibody; Ig, immunoglobulin; IU/ml, international unit per ml. Blue bars/dots indicate IgG titers ≥11 IU/ml (protective), whereas red bars/dots show titers below this threshold.

Serologic analysis revealed generally homogeneous antibody titers across age groups (Figure 1). Among the five under-immunized individuals (IgG <11 IU/ml), three were younger than 35 years (26, 27, and 29 years). No significant associations were found between immunity status and body mass index (P = 0.67), comorbidities (31.8% vs 37.5%, P = 0.65), regular medication use (9.8% vs 6.2%, P = 0.64), or alcohol consumption (50.8% vs 50%, P = 0.95).

### Acceptance of anti-MMR vaccine by the HBM

The analysis of the HBM revealed that perceived benefits had the highest median score (median = 8, IQR = 7-10), followed by perceived susceptibility (median = 8, IQR = 6-10) and perceived severity (median = 8, IQR = 6-10). Perceived barriers had the lowest median score (median = 4, IQR = 2-6). No statistically significant differences were observed between the immunized and non-immunized cohorts in the scores among the HBM domains (Table 4).

**Table 4:** Health belief model of HCPs.

	Total (n = 148)^a^	HCWs immunized against measles (n = 116, 95.1%)	HCWs not immunized against measles (n = 5, 4.1%)	*P*
Perceived susceptibility				
Median score (IQR)	8 (6-10)	8 (6-10)	8 (8-8)	0.31
Low (<median)	70 (57.9%)	66 (56.9%)	4 (80%)	0.3
High (>median)	51 (42.1%)	50 (43.1%)	1 (20%)	
Perceived severity				
Median score (IQR)	8 (6-10)	8 (6-10)	8 (8-8)	0.4
Low (<median)	75 (62%)	71 (61.2%)	4 (80%)	0.39
High (>median)	46 (38%)	45 (38.8%)	1 (20%)	
Perceived barriers				
Median score (IQR)	4 (2-6)	4 (2-6)	4 (4-6)	0.92
Low (<median)	70 (57.8%)	67 (57.8%)	3 (60%)	0.9
High (>median)	51 (42.2%)	49 (42.2%)	2 (40%)	
Perceived benefits				
Median score (IQR)	8 (7-10)	8 (7-10)	8 (8-9)	0.69
Low (<median)	62 (51.2%)	59 (50.9%)	3 (60%)	0.69
High (>median)	59 (48.8%)	57 (49.1%)	2 (40%)	

HCPs, health care professionalss; IQR, interquartile range.

a Among the 148 health care professionals recruited, 27 declined to undergo blood sampling for serological analysis.

The multivariate analysis (Table 5) showed that smoking status was significantly associated with lower measles immunization of HCPs (adjusted odds ratio = 13.3; 95% CI= 1.07-166.2; *P* <0.04), whereas sex, age, body mass index, professional category, and HBM domains were not significantly associated.

**Table 5:** Univariable and multivariable analysis of factors associated with low immunization status.

	Univariable analysis			Multivariable analysis		
	OR crude	CI 95%	*P*	OR adjusted	CI 95%	*P*
Male vs female	1.33	0.14-12.41	0.8	1.61	0.11-23.52	0.72
Age per year increase	0.33	0.03-3.04	0.32	0.38	0.03-4.66	0.45
Obese vs normal weight	3.86	0.42-35.62	0.23	3.79	0.34-42.27	0.28
Physician vs other HCPs	2.69	0.42-17.08	0.29	11.23	0.45-279.26	0.14
Years of working experience 5-10 working years vs <5 years	2.6	0.2-31	0.44	-	-	-
>10 working years vs <5 years	3.3	0.28-39	0.34	-	-	-
>20 working years vs <5 years	5.76	0.5-67.6	1.4	-	-	-
Comorbidities: yes vs no	0.98	0.73-1.33	0.94	-	-	-
Drug consumption: yes vs no	1	-	-	-	-	-
Smoking: active smoker vs never smoked	4.11	0.65-25.78	0.13	13.3	1.07-166.2	0.04
Alcohol consumption: yes vs no	0.16	0.01-1.5	0.1	-	-	-
Recommendation of the vaccine: yes vs no	0.85	0.09-8.22	0.89	-	-	-
Higher vs lower perceived susceptibility	0.33	0.035-3.04	0.33	0.64	0.03-12.58	0.77
Higher vs lower perceived severity	0.39	0.04-3.64	0.41	0.12	0-3.89	0.23
Higher vs lower perceived barriers	0.91	0.14-5.66	0.92	0.66	0.08-5.14	0.69
Higher vs lower perceived benefits	0.69	0.11-4.28	0.69	0.71	0.04-11.87	0.8

CI, interval of confidence; HCP, health care professional; OR, odds ratio.

## Discussion

By late 2021, European health authorities intensified immunization efforts to address underreported measles circulation, worsened by declining vaccination rates during the COVID-19 pandemic [11]. Since early 2023, Italy has seen a steady rise in measles cases, exceeding 1,000 in 2024—the highest among 10 European countries [3]. Key drivers include suboptimal second dose coverage (<95%) and imported cases. In response, the Italian National Vaccination Prevention Plan 2023-2025 prioritizes reducing vaccine-preventable diseases through strengthened surveillance, public education, fostering vaccine confidence, and specialized HCP training [5].

This study found 96% seroprevalence of protective anti-measles IgG among HCPs, consistent with the World Health Organization’s 95% threshold and supporting herd immunity [3]. Comparable coverage has been reported in Italian regions, ranging from 84.8% to 97.7% [[19], [20], [21], [22], [23]]. Given measles’ high contagion and severity in health care settings, vaccinating susceptible HCPs is crucial to prevent nosocomial transmission, particularly, in high-risk wards [24]. In 2017, over 4,400 cases occurred among infants aged under 1 year and 7% of HCPs were infected, including three deaths and two encephalitis cases [25]. In 2024, the Italian National Institute of Health reported 1.64 cases per million in health care settings [3], reaffirming vaccination as the only effective preventive measure [3]. These findings are consistent with European evidence showing that ensuring measles immunity among HCPs is essential to preventing nosocomial outbreaks and protecting vulnerable patient groups, particularly, in high-risk hospital settings [26,27].

Logistic regression showed a significant association between smoking and reduced immunoglobulin levels, suggesting behavioral risk factors may weaken immunity. Despite the small sample, these findings align with previous evidence linking smoking to impaired innate and adaptive immune responses [[28], [29], [30]], warranting larger studies to confirm and guide preventive strategies. Since 1968, studies have shown reduced immune activity among smokers, with later evidence confirming lower IgG and IgA levels [[31], [32], [33], [34], [35]]. A recent meta-analysis linked smoking to greater infection risk and immune disorders [30] via impaired immunoglobulin production, altered cytokines, and epigenetic changes [28]. Although tobacco’s immunosuppressive effects on vaccine responses, such as influenza, are documented [31], no studies have specifically examined measles immunity, highlighting the need for further research.

Consistent with other Italian studies [20], no significant differences in antibody levels emerged across age groups or genders, although immunity was slightly higher in males (97.5%) than in females (95.3%). MMR-induced immunity may wane over time, with antibody titers falling below protective levels ∼15 years post-vaccination unless boosted by natural exposure [36]. Among unvaccinated HCPs, most non-immune individuals were young women aged under 33 years (60%), likely reflecting Italian Decree No. 73/2017, which mandated vaccination only for those born after 2000 [7]. Such immunity gaps are concerning in health care, where HCPs face high exposure risks and may transmit measles to vulnerable patients. Importantly, MMR vaccination in young female HCPs also protects against rubella, a crucial aspect in preventing congenital rubella syndrome in pregnant women. Suboptimal vaccination coverage among HCPs and medical students has been documented across Europe, particularly, in high-risk settings [37,38].

Among non-immunized HCPs, 60% worked in obstetrics, representing 8% of department participants. Although based on a small sample, this suggests workplace-related factors, limited vaccine awareness, or risk perception issues. Non-immune HCPs in settings with vulnerable patients, such as pregnant women, raise concerns and underscore the need for targeted awareness campaigns and vaccination strategies. This is especially relevant in Italy, where measles vaccination for HCPs is strongly recommended but not mandatory.

In response to similar concerns, in 2018 the Emilia-Romagna (Regional decision n. 351 of March 12, 2018) and Puglia (Regional decision n. 27 of June 21, 2018) regional health authorities introduced specific regulations requiring the assessment of measles immunity among HCPs during recruitment. Under these measures, susceptible workers who declined vaccination were reassigned to low-risk departments to prevent exposure in high-risk areas such as oncology and hematology. Occupational physicians could similarly assess HCPs’ immune status during mandatory health surveillance (Italian Decree 81/08) [39]. Italian law provides effective vaccines for susceptible workers, administered by occupational physicians [39]. Given waning immunity, serological status should be evaluated at recruitment and 10-15 years post-vaccination [11], ensuring HCPs’ protection and reducing nosocomial transmission. Similar concerns have been raised at the European level, where several countries have strengthened vaccination policies for health care personnel to mitigate the risk of measles transmission in hospitals [26]. Evidence from recent outbreaks further highlights that unvaccinated HCPs are particularly affected, contributing to nosocomial spread [27].

Finally, class I obesity was more frequent among non-immunized participants (25% vs 14.4%), possibly reflecting behavioral or metabolic influences on immunity. Other comorbidities and medication use showed modest, non-significant differences, whereas alcohol consumption was comparable, suggesting no clear association with measles immunity. No significant differences emerged between immunized and non-immunized HCPs across HBM domains, although concerning attitudes persist. A previous regional study reported high perceived barriers in 57.4% of HCPs, consistent with our findings (60%) [40]. Among non-immunized HCPs, 42.6% underestimated measles severity, 20.4% questioned vaccine benefits, and 24.1% did not view recommending vaccination as their responsibility [40]. These results highlight misperceptions and a lack of awareness, likely linked to inadequate immunization training. Strengthening educational initiatives is essential to improve HCPs’ understanding of their preventive role and the importance of vaccination.

This study has several limitations that need to be underlined. First, the survey included HCPs from only two hospitals in the province of Enna, limiting the generalizability of findings. The small size of some subgroups, such as smokers, restricts interpretation, making the results exploratory and requiring confirmation in larger studies. Exposure risk assessment was also affected because departments such as emergency, infectious diseases, and pediatrics, known for higher exposure due to patient profiles, were under-represented. Recall bias in questionnaire responses may have further underestimated exposures. Despite these constraints, the study provides valuable serological data on HCPs’ immunization status during a period of rising measles incidence in Europe and the United States, contributing to the ongoing debate on this urgent public health issue.

## Conclusion

This study indicates that measles immunity among HCPs is generally adequate, although immunity gaps persist among younger adults, reflecting potential limitations in previous vaccination policies. Although no substantial differences emerged across genders or professional roles, non-immune individuals were more frequently found among nursing and health care support staff, highlighting the need for targeted educational and immunization initiatives. The significant association between smoking status and lower measles immunization suggests aspects requiring further investigation. Overall, the findings underscore the importance of enhanced surveillance and focused vaccination strategies, confirming the need for larger multicentric studies to validate these observations and more comprehensively explore determinants of vaccine adherence.

## Author contributions

Conceptualization: A.D., R.V., and V.E.; methodology: R.V., M.B., C.F., and V.E.; software: R.V.; validation: V.E., S.P., L.B., and C.F.; formal analysis: R.V.; investigation: A.D.; resources: V.E., L.B., and R.V.; data curation: C.F., S.P., B.M.; writing—original draft preparation: A.D.; writing—review and editing: R.V., C.F., M.B., L.B., and V.E.; visualization: A.D., S.P., and L.B.; supervision: R.V., M.B., and V.E.; project administration: R.V., M.B., and V.E.; funding acquisition: V.E.

## Institutional review board statement

The study was approved by the Ethical Committee of University of Enna Kore at the July 24, 2025 meeting.

## Informed consent statement

Informed consent was obtained from all subjects involved in the study.

## Data availability statement

Data will be available upon a motivated request to the corresponding author.

## Declaration of competing interest

The authors have no competing interests to declare.

## Footnotes

1 The heading of Figure 1 is "Box plot with measles IgG antibody index by age for HCWs." and should be placed in the appropriate position deleting it from subheading.
